# A_2B_ adenosine receptor inhibition by the dihydropyridine calcium channel blocker nifedipine involves colonic fluid secretion

**DOI:** 10.1038/s41598-020-60147-7

**Published:** 2020-02-26

**Authors:** Teita Asano, Yuto Noda, Ken-Ichiro Tanaka, Naoki Yamakawa, Mitsuhito Wada, Tadaaki Mashimo, Yoshifumi Fukunishi, Tohru Mizushima, Mitsuko Takenaga

**Affiliations:** 1 Institute of Medical Science, St. Marianna University School of Medicine, 2-16-1, Sugao, Miyamae-ku Kawasaki, 216-8512 Japan; 2 LTT Bio-Pharma Co., Ltd, Shiodome Building 3F, 1-2-20 Kaigan, Minato-ku Tokyo, 105-0022 Japan; 3 Research Institute of Pharmaceutical Sciences, Faculty of Pharmacy, Musashino University, 1-1-20, Shin-machi, Nishi-Tokyo, 202-8585 Japan; 4 School of Pharmacy, Shujitsu University, 1-6-1, Nishi-kawahara, Naka-ku Okayama, 703-8516 Japan; 5 Technology Research Association for Next Generation Natural Products Chemistry, 2-3-26, Aomi, Koto-ku Tokyo, 135-0064 Japan; 6 Molecular Profiling Research Center for Drug Discovery (molprof), National Institute of Advanced Industrial Science and Technology (AIST), 2-3-26, Aomi, Koto-ku Tokyo, 135-0064 Japan; 7 IMSBIO Co., Ltd., Owl Tower, 4-21-1, Higashi-Ikebukuro, Toshima-ku Tokyo, 170-0013 Japan

**Keywords:** Biophysical chemistry, Pharmacology, Gastrointestinal diseases

## Abstract

The adenosine A_2B_ receptor is a critical protein in intestinal water secretion. In the present study, we screened compound libraries to identify inhibitors of the A_2B_ receptor and evaluated their effect on adenosine-induced intestinal fluid secretion. The screening identified the dihydropyridine calcium antagonists nifedipine and nisoldipine. Their respective affinities for the A_2B_ receptor (*K*_i_ value) were 886 and 1,399 nM. Nifedipine and nisoldipine, but not amlodipine or nitrendipine, inhibited both calcium mobilization and adenosine-induced cAMP accumulation in cell lines. Moreover, adenosine injection into the lumen significantly increased fluid volume in the colonic loop of wild-type mice but not A_2B_ receptor-deficient mice. PSB-1115, a selective A_2B_ receptor antagonist, and nifedipine prevented elevated adenosine-stimulated fluid secretion in mice. Our results may provide useful insights into the structure–activity relationship of dihydropyridines for A_2B_ receptor. As colonic fluid secretion by adenosine seems to rely predominantly on the A_2B_ receptor, nifedipine could be a therapeutic candidate for diarrhoea-related diseases.

## Introduction

Adenosine is an important mediator of multiple functions, such as intestinal secretion, contraction, inflammation, and sensation in the gastrointestinal tract^1–3^. Among adenosine receptors, the A_2B_ receptor is highly expressed in the colon and has critical roles in pathological conditions^4^. In general, adenosine levels and A_2B_ receptor expression are low under normal conditions, but increase in response to ischemia, inflammation, and tissue injuries. The activated A_2B_ receptor stimulates intestinal water secretion and sensation or modulates inflammatory response and colonic motility^5,6^. As a result, it is associated with the development of gastrointestinal diseases, such as irritable bowel syndrome (IBS), secretary diarrhoea, and inflammatory bowel disease^7,8^. Therefore, this receptor has attracted substantial attention as a therapeutic target. Although pharmacological and molecular tools, including genetically modified mouse models, have revealed multiple functions of the A_2B_ receptor, such as immunomodulation, relaxation of smooth muscle, and intestinal secretion^5,9^, our understanding of its biology remains unclear.

Diarrhoea is caused by bacterial and viral infections, inflammatory processes, drugs, genetic disorders, and abnormal intestinal secretion or electrolyte absorption^10,11^. However, the pathophysiology of diarrhoea is not fully understood. Chloride secretion into the lumen following activation of the cystic fibrosis transmembrane conductance regulator (CFTR) channel in intestinal epithelial cells plays a crucial role in secretory diarrhoea^1,12^. Several studies suggest that A_2B_ receptor activation causes fluid secretion into the lumen. *In vitro* studies using colonic epithelial cells have demonstrated that adenosine increases luminal water volume through apical or basolateral A_2B_ receptors^13,14^. Although both cAMP activation via Gs proteins and the intracellular calcium pathway contribute to chloride or water secretion in epithelial cells, chloride release is most likely due to cAMP elevation following A_2B_ receptor activation^1,15^. A recent report has shown that A_2B_ receptor-mediated direct activation of the CFTR channel is associated with water secretion in epithelial cells^12^. Nevertheless, evidence that the A_2B_ receptor modulates fluid secretion has remained limited, in part because of a lack of appropriate pharmacological agents or genetically modified mice.

Recently, we reported that blockade of the A_2B_ receptor could be beneficial for diarrhoea-predominant IBS^8^. Inhibition of the receptor by pharmacological agents ameliorated colonic hypersensitivity and stress-induced defecation in rodent models of IBS. In the present study, we screened a library of approved medicinal compounds for candidate drugs that could suppress adenosine A_2B_ receptor activation. We also determined the role of various adenosine receptor subtypes involved in colonic fluid secretion induced by luminal adenosine and examined the effect of the candidate drug on fluid secretion in mice.

## Results

### Identification of nifedipine through screening for adenosine A2B receptor binding compounds

**Figure 1:**
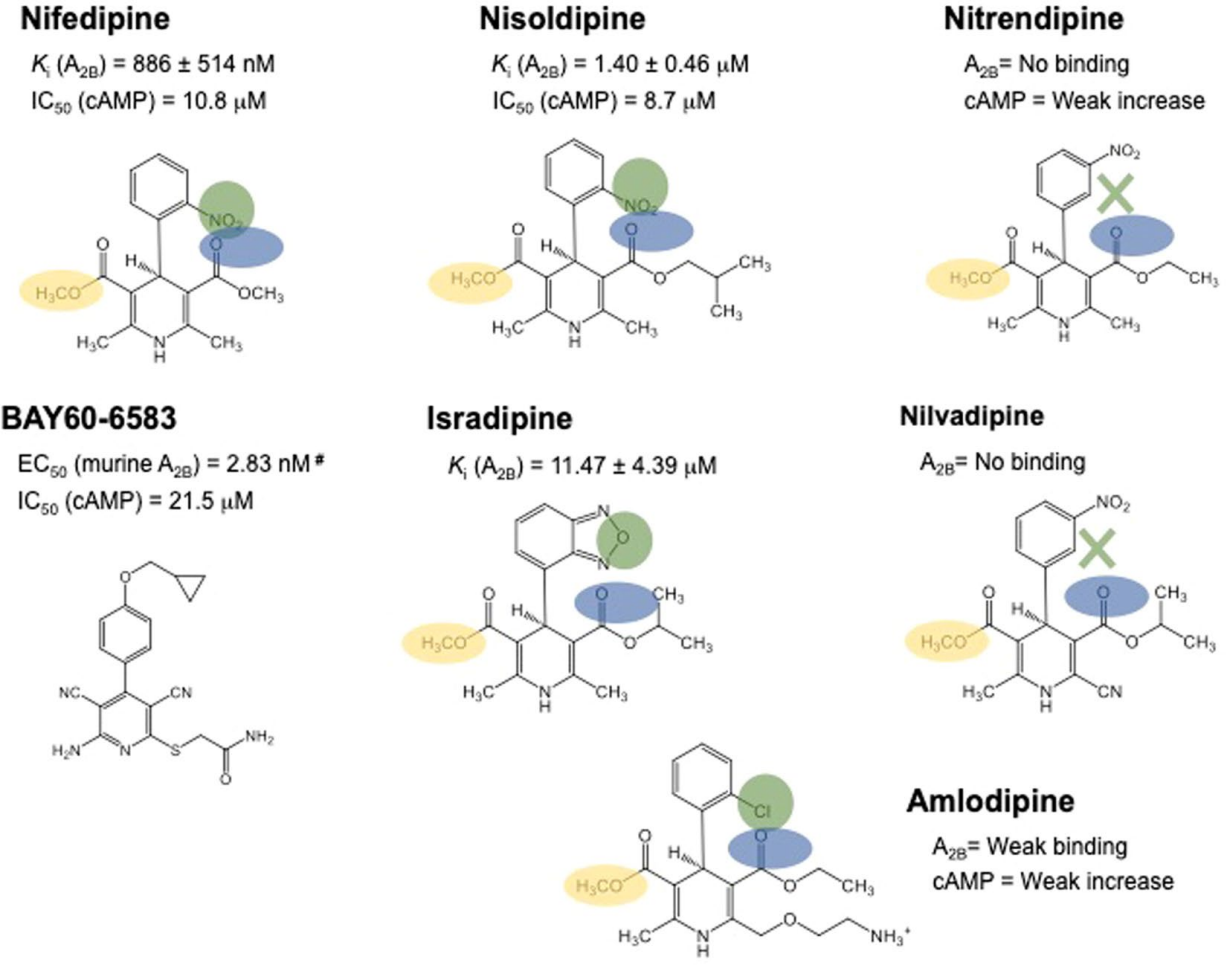
Summary of the compounds. ‘*K*_i_ (A_2B_)’ values are the [^3^H]-DPCPX binding assay results and ‘cAMP’ values are the adenosine-induced cAMP assay results. Pharmacophore analysis of the compounds was performed. Green, blue, and orange circles represent the hydrogen donor site binding His280^7.43^, a hydrogen donor with side-chain binding to Tyr10^1.35^, and the common –OCH_3_ group close to Ser279^7.42^, respectively. Green cross ‘X’ represents the mismatch between the ligand NO_2_ group and the pharmacophore. ^#^, the values of BAY60-6583 were adopted from van der Hoeven *et al*.^44^.

**Figure 2:**
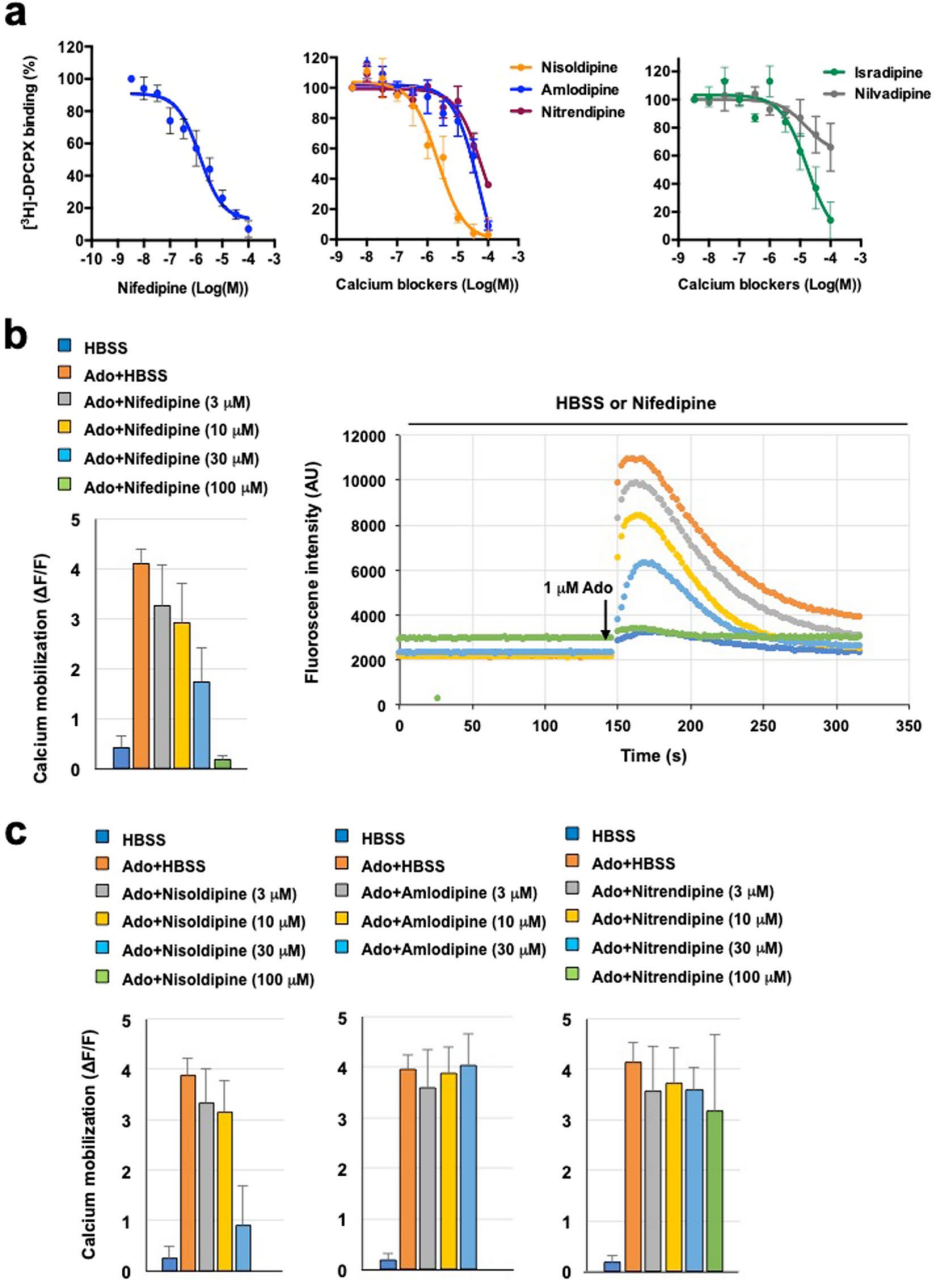
Binding affinity of dihydropyridine calcium blockers for the A_2B_ receptor and effect on calcium mobilization. (**a**) Curves generated from competition displacement experiments in HEK293 cells expressing human adenosine A_2B_ receptor in the presence of [^3^H]-DPCPX and various calcium antagonists (nifedipine, nisoldipine, amlodipine, isradipine, nitrendipine, and nilvadipine). Data represent the average of three independent experiments. (**b**) Calcium mobilization assay in CHO cells stably expressing both human adenosine A_2B_ receptor and Gα15 protein and incubated with various concentrations of nifedipine. Cells were stimulated by injection with 1 µM adenosine (Ado) solution or HBSS. Calcium mobilization (∆F/F) was calculated based on fluorescence intensity readings. (**c**) Calcium mobilization assay in the presence of various concentrations of nisoldipine, amlodipine, and nitrendipine. All data are expressed as mean ± SEM. [^3^H]-DPCPX, 8-cyclopentyl-1,3-dipropylxanthine, [dipropyl-2,3-^3^H(N)].

Screening of a compound library identified 12 candidate drugs with binding affinity for the A_2B_ receptor (Supplementary Table 1). Of these, three were already known, four had a xanthine-like structure, and two were dihydropyridine calcium channel blockers. We focused on the latter, nifedipine and nisoldipine, because dihydropyridine is similar to the structure of BAY60-6583, a known adenosine A_2B_ receptor agonist (Fig. 1). Nifedipine inhibited [^3^H]-DPCPX binding to the A_2B_ receptor membrane in a dose-dependent manner and its *K*_i_ value was 886 ± 514 nM (Fig. 2a). We also examined the binding activity of other dihydropyridine calcium channel blockers and Fig. 1 summarizes the results. Nisoldipine but not nitrendipine and nilvadipine significantly suppressed [^3^H]-DPCPX binding, whereas isradipine and amlodipine exhibited only weak binding to the A_2B_ receptor (Fig. 2a). The *K*_i_ values of nisoldipine and isradipine were 1.40 ± 0.46 µM and 11.47 ± 4.39 µM, respectively. We confirmed the effect of nifedipine on adenosine-induced calcium mobilization in CHO cells stably expressing the A_2B_ receptor. Dose-dependent calcium mobilization by adenosine was observed, with fluorescence being nearly maximal with 10 µM of adenosine (data not shown). As shown in Fig. 2b,c, adenosine caused immediate calcium mobilization while nifedipine dose-dependently blocked it but did not stimulate it. Nisoldipine had a similar effect, whereas amlodipine and nitrendipine did not. The IC_50_ values of nifedipine and nisoldipine were 42.4 µM and 34.0 µM, respectively.

### Effect of dihydropyridine calcium blockers on adenosine-induced cAMP production in intestinal epithelial cells

**Figure 3:**
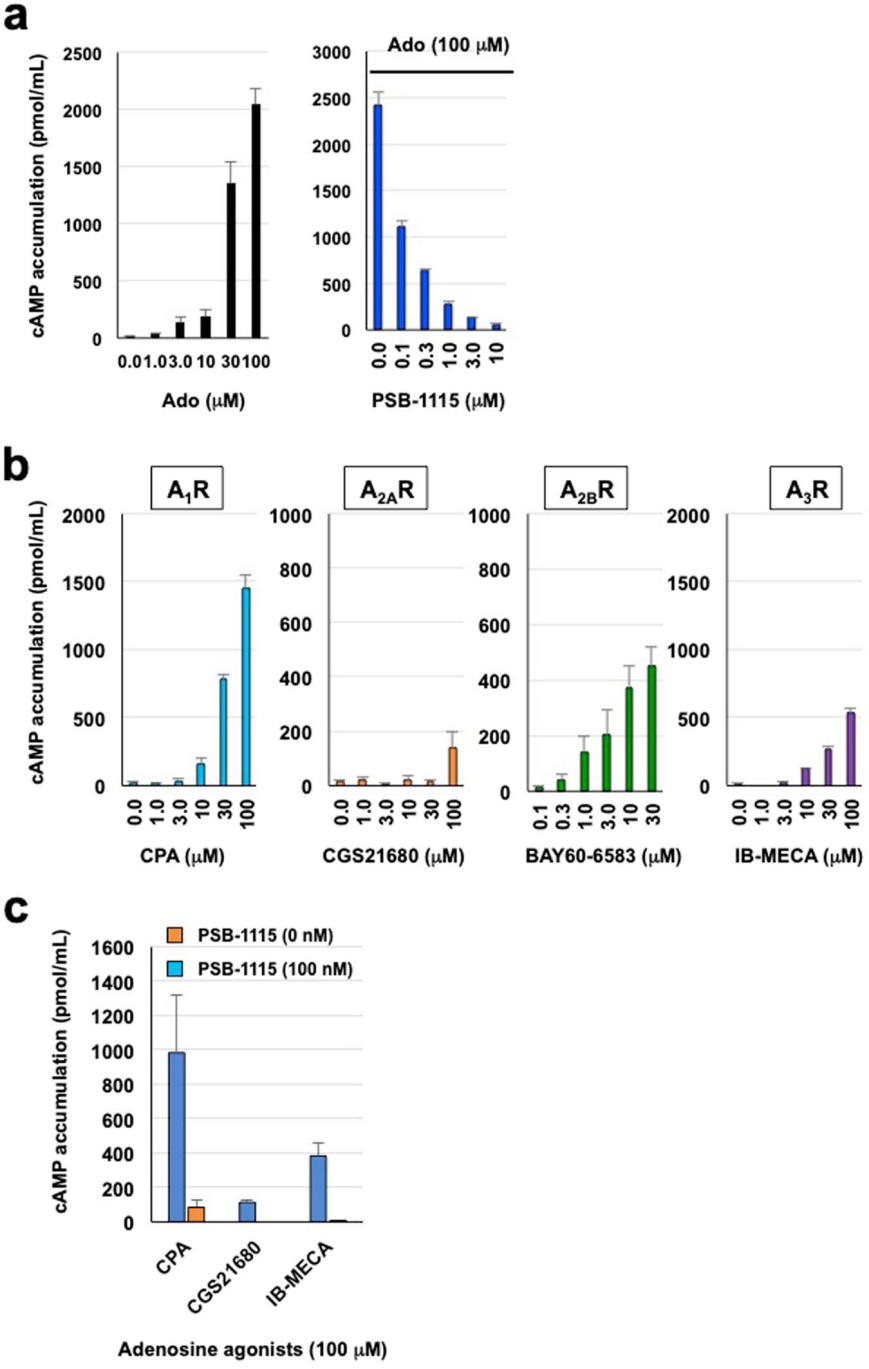
Responsiveness of cAMP production to adenosine receptor agonists in T84 cells. (**a**) Intracellular cAMP levels in confluent epithelial T84 cells incubated with various concentrations of either adenosine (Ado) or the selective A_2B_ receptor antagonist PSB-1115 for 15 min. (**b**) Intracellular cAMP levels in confluent T84 cells incubated with various concentrations of the selective receptor agonists CPA (A_1_R), CGS21680 (A_2A_R), BAY60-6583 (A_2B_R), or IB-MECA (A_3_R). (**c**) Intracellular cAMP levels in confluent T84 cells incubated with various concentrations of selective receptor agonists (100 μM) and the A_2B_ receptor antagonist PSB-1115 (100 nM). All data are expressed as mean ± SEM.

Next, we examined the effect of nifedipine on intracellular cAMP levels in colonic epithelial T84 cells. Fluid secretion and dominant A_2B_ receptor expression have already been reported in this cell line^13^. First, we characterized the cells’ cAMP response to adenosine agonists. Adenosine or BAY60-6583 (a specific A_2B_ receptor agonist) induced cAMP accumulation in a significant dose-dependent manner, whereas a selective A_2B_ antagonist, PSB-1115, suppressed adenosine-produced cAMP elevation (IC_50_ = 84.0 nM) (Fig. 3a,b). Pharmacological analysis using specific agonists showed that CPA (A_1_ receptor) increased cAMP levels in T84 cells and both BAY60-6583 (A_2B_ receptor) and IB-MECA (A_3_ receptor) induced modest stimulation, whereas CGS21680 (A_2A_ receptor) had only a minor effect (Fig. 3b). cAMP elevation by CPA, CGS21680, and IB-MECA was completely blocked by PSB-1115 (Fig. 3c), suggesting that the observed effect was mediated by their non-specific binding to the A_2B_ receptor. Thus, cAMP accumulation induced by adenosine in T84 cells likely involves the A_2B_ receptor.

**Figure 4:**
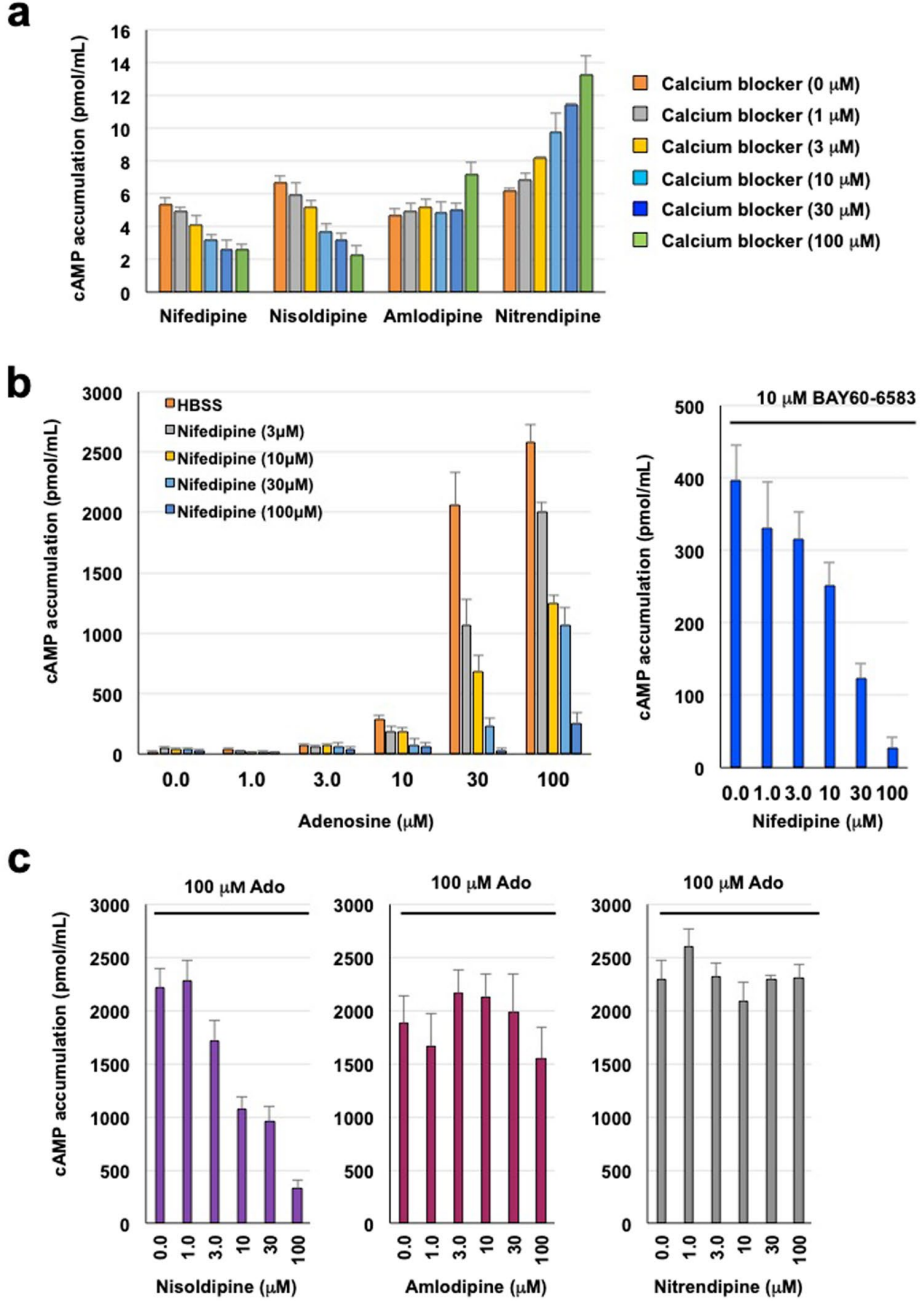
Effect of calcium blockers on adenosine-induced cAMP accumulation in T84 cells. (**a**) Intracellular cAMP levels in confluent T84 cells incubated with various concentrations of nifedipine, nisoldipine, amlodipine, or nitrendipine. (**b**) Intracellular cAMP levels in the presence of varying concentrations of adenosine (0–100 μM) or 10 µM BAY60-6583 for 15 min. (**c**) Intracellular cAMP levels in the presence of 100 µM adenosine (Ado) and various concentrations of nisoldipine, amlodipine, or nitrendipine. All data are expressed as mean ± SEM.

We then examined the effect of dihydropyridine calcium blockers on cAMP response in T84 cells. In the absence of adenosine, both nifedipine and nisoldipine slightly decreased intracellular cAMP levels, nitrendipine caused an increase, and amlodipine had no significant effect (Fig. 4a). In addition, nifedipine dose-dependently inhibited cAMP accumulation (Fig. 4b) induced by either adenosine (IC_50_ = 10.8 µM) or BAY60-6583 (IC_50_ = 21.5 µM). Nisoldipine, but not amlodipine or nitrendipine, exhibited similar dose-dependent inhibition of adenosine-induced cAMP levels (IC_50_ = 8.7 µM) (Fig. 4c). The results are summarized in Fig. 1. To determine whether nifedipine decreases intracellular cAMP levels independent of its A_2B_ receptor blockade, we examined the effect of nifedipine on an adenylate cyclase activator, forskolin, which induced increases in cAMP levels. We observed no influence by nifedipine on elevated cAMP by the compounds, suggesting that adenosine-stimulated cAMP elevation is dependent on the A_2B_ receptor (data not shown).

### Effect of nifedipine on adenosine-induced colonic fluid secretion in mice

**Figure 5:**
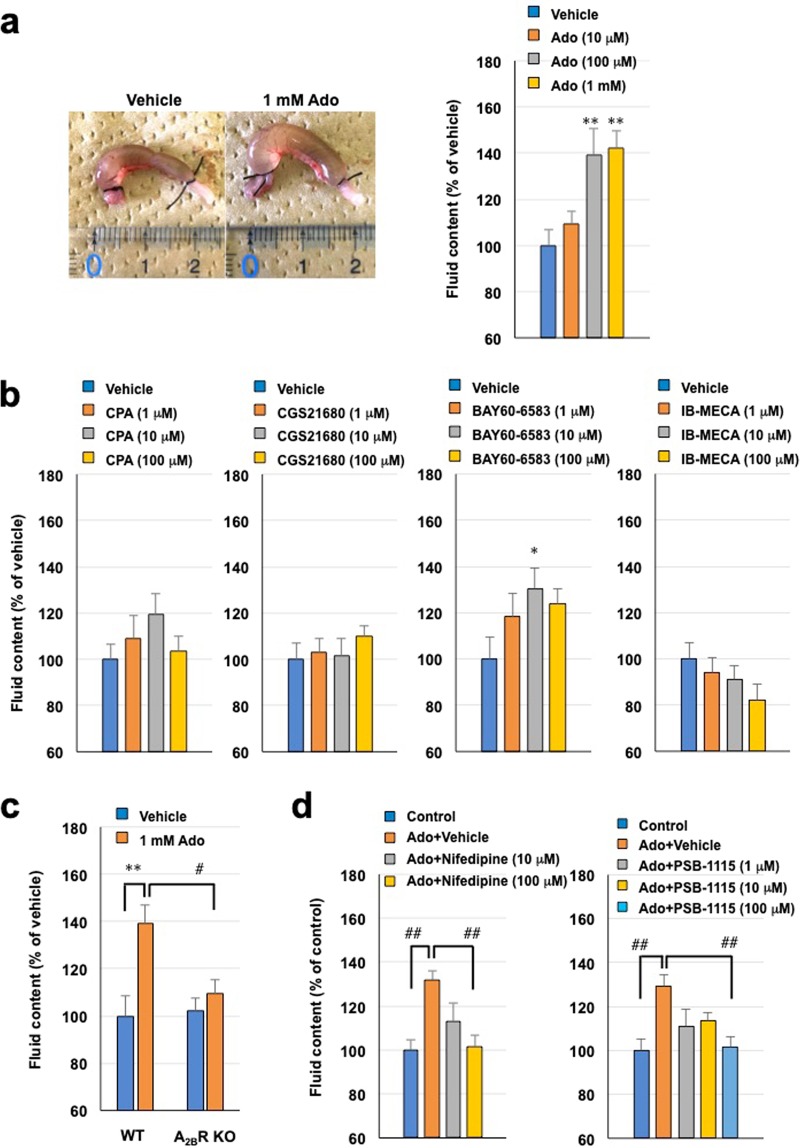
Involvement of adenosine A_2B_ receptor and effectiveness of nifedipine in colonic fluid secretion in mice. (**a**) Representative image of colonic loop treated with vehicle (saline containing 2.5% DMSO) or 1 mM adenosine (Ado), and colonic fluid volume following injection of various doses of adenosine. (**b**) Effect of the selective adenosine receptor agonists CPA (A_1_R), CGS21680 (A_2A_R), BAY60-6583 (A_2B_R), and IB-MECA (A_3_R) on colonic fluid volume in C57/BL6 mice. (**c**) Adenosine-induced colonic fluid secretion in C57/BL6 and A_2B_ receptor-deficient mice. (**d**) Effect of nifedipine or PSB-1115 on adenosine (1 mM)-induced colonic fluid secretion in mice. All data are expressed as mean ± SEM. ^*^P < 0.05 or ^**^P < 0.01 (*vs*. vehicle). ^#^*P* < 0.05 or ^##^*P* < 0.01 (*vs* Ado in wild-type).

Finally, we investigated whether the A_2B_ receptor stimulated intestinal fluid secretion and whether nifedipine suppressed it *in vivo*. First, we characterized fluid secretion induced by luminal adenosine in C57BL/6 mice. As shown in Fig. 5a, adenosine injection into the colonic lumen increased fluid content in a dose-dependent manner. Analysis of subtype-specific agonists revealed that only the adenosine A_2B_ receptor agonist BAY60-6583 significantly increased luminal fluid content (Fig. 5b). Confirming this point, increased fluid production by luminal adenosine was absent from A_2B_ receptor-knockout mice (Fig. 5c). These results suggest that stimulation of fluid secretion by luminal adenosine is dependent on the A_2B_ receptor, and it was suppressed by nifedipine and PSB-1115 (Fig. 5d).

## Discussion

Increased intestinal fluid secretion plays an important role in diarrhoea-related diseases such as IBS, and infection- or drug-induced diarrhoea^10,11^. Several studies have reported an association between adenosine A_2B_ receptor and intestinal fluid secretion. Inhibition of the A_2B_ receptor has been shown to be effective against IBS with diarrhoea in rodents^8^. To identify candidate A_2B_ receptor inhibitors, we screened a library of medicines in clinical use. As their pharmacokinetics, pharmacodynamics, metabolism, tolerability, and toxicity are already known in humans, they can be relatively easily included in a pilot study^16^. Using a competitive binding assay, we identified four drugs with xanthine-like structures and three known A_2B_ receptor binding compounds, including aminophylline, confirming the specificity of the screening (Supplementary Table 1). Considering that A_2B_ receptor antagonists without a xanthine backbone had not been reported previously, we focused on two dihydropyridine calcium antagonists, nifedipine and nisoldipine, whose structure is similar to BAY60-6583, a selective A_2B_ receptor agonist (Fig. 1). The inhibitory action of nifedipine and nisoldipine could reveal important structure–activity information.

Unlike other calcium antagonists (e.g., nitrendipine, amlodipine, nilvadipine, and isradipine), nifedipine and nisoldipine exhibited significant binding affinity for the A_2B_ receptor. This property may be explained by the presence of an ortho-nitric oxide substitute on the benzene ring of nifedipine and nisoldipine that is absent from other compounds (Fig. 1). This nitric oxide group might be critical in preventing the binding of adenosine to the A_2B_ receptor. To confirm the competition assay results, we performed a calcium mobilization test in CHO cells stably expressing the A_2B_ receptor. Both nifedipine and nisoldipine inhibited but did not stimulate this calcium response, with the former exhibiting a stronger inhibitory action, in line with the competitive binding assay results. This result also suggested that both calcium blockers have antagonistic activity for A_2B_ receptor.

As a mechanism of colonic fluid secretion by A_2B_ receptor activation, intracellular cAMP elevation by Gs protein and mobilisation of intracellular calcium by Gq protein have been suggested^1^. Nichols *et al*. recently reported that cAMP elevation but not calcium mobilisation mediated by Gq-protein in T84 colonic epithelial cells predominantly contributes to secretary activity into the lumen through CFTR channel activation^17^. Therefore, we confirmed the antagonistic activities of nifedipine and nisoldipine on adenosine- or BAY60-6583-dependent activation in T84 cells using a cAMP accumulation assay. Again, amlodipine and nitrendipine showed no effect, consistent with the calcium assay results. We also determined a weak inhibition of intracellular cAMP levels by nifedipine and nisoldipine in the absence of adenosine stimulation. Amlodipine and nitrendipine did not inhibit adenosine-induced cAMP levels and showed a weak increase in cAMP without adenosine stimulation.

While the expression profiles of adenosine receptors and intestinal fluid secretion have been extensively characterized in T84 cells^18^, responsiveness to specific agonists for each adenosine receptor has not been examined. Here, we investigated the cAMP response to activation of each adenosine receptor. We speculated that cAMP production by adenosine receptor agonists originated predominantly from A_2B_ receptor activation in the cells. In Fig. 3b, CPA, CGS21680, and IB-MECA caused cAMP elevation at a concentration of nearly 100 µM. However, we consider that these results are derived from non-specific A_2B_ receptor binding of these agonists, because the *K*_i_ values of CPA, CGS21680, and IB-MECA for A_2B_ receptors are 18,600 nM,>10,000 nM, and 11,000 nM, respectively^19^. In addition, elevated intracellular cAMP levels by these agonists were blocked by a selective A_2B_ antagonist, PSB-1115 (Fig. 3c). Furthermore, a previous study showed low or undetectable expression of A_1_ and A_3_ receptors in T84 cells^18^. Thus, adenosine- or BAY60-6583-induced cAMP accumulation may be predominantly mediated by A_2B_ receptor. Although we do not evaluate this in normal intestinal epithelial cells and cannot fully exclude the association with A_2A_ receptor, activation of A_2B_ receptor by binding of extracellular adenosine might cause an increase in intracellular cAMP levels through Gs protein, followed by CFTR channel activation in intestinal epithelial cells, leading to fluid secretion.

**Figure 6:**
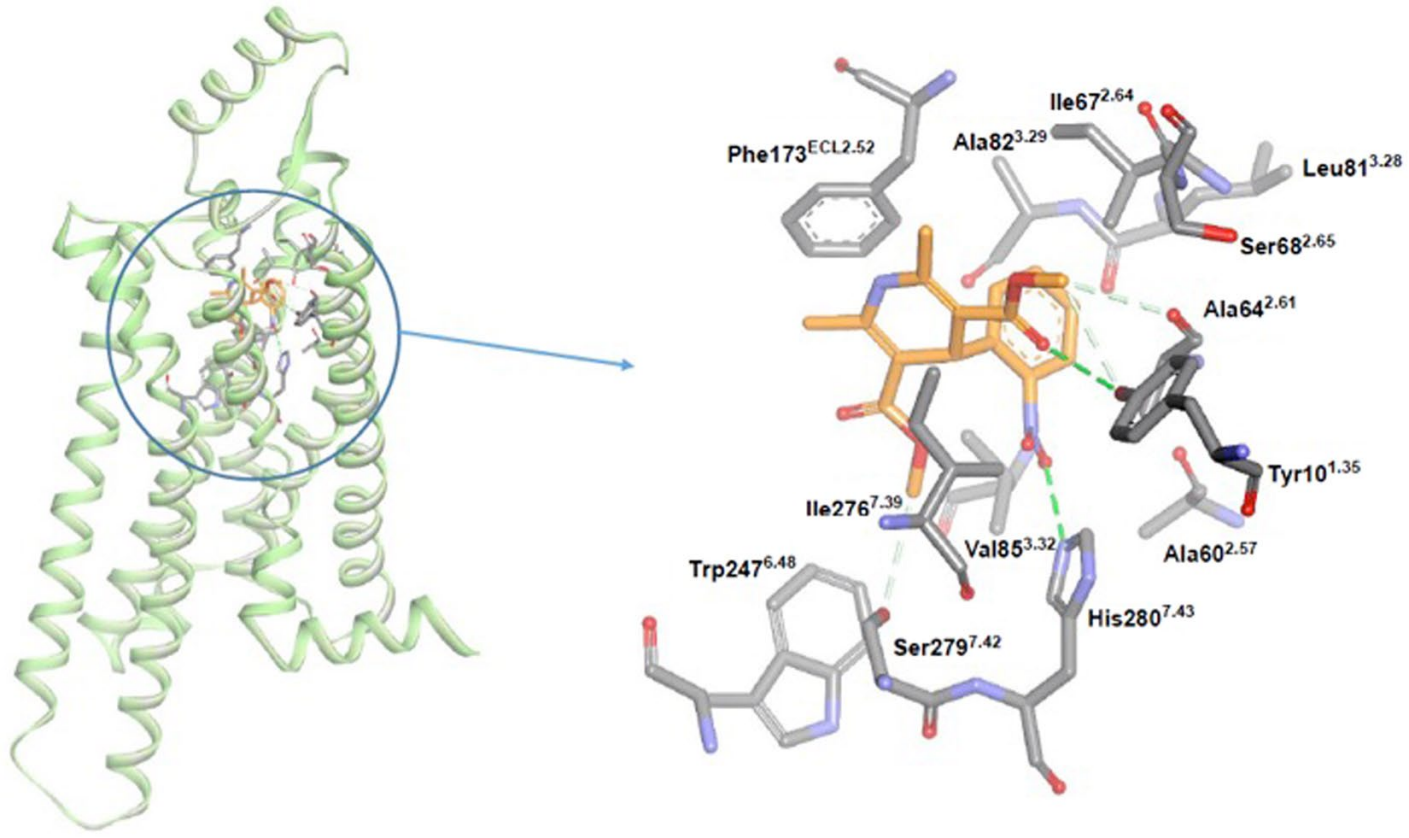
Docking model of the interaction between human adenosine A_2B_ receptor and nifedipine. Nifedipine is shown as a stick model, with carbon coloured in yellow, nitrogen in blue, hydrogen in white, and oxygen in red. Broken lines connecting Tyr10^1.35^, Ala64^2.61^, His280^7.42^, and Ser279^7.42^ represent possible hydrogen bonds (or CH…O interactions) with nifedipine. The distances of nifedipine from Tyr10^1.35^, Ala64^2.61^, Phe173^ECL2.52^, His280^7.42^, and Ser279^7.42^ were calculated to be 3.38 Å, 3.42 Å, 3.35 Å, 2.86 Å, and 3.54 Å, respectively, without taking account of hydrogens. The dihydropyridine of nifedipine can form a π–π stacking interaction with Phe173^ECL2.52^.

Moreover, we observed that both adenosine- and BAY60-6583-stimulated cAMP increases were blocked by nifedipine. The latter may bind to an active site shared by both agonists. Site-directed mutagenesis suggests that Phe173^2.52^ and Trp247^6.48^ on the A_2B_ receptor are important for BAY60-6583 and adenosine binding^20,21^. The present docking study supported the above mutagenesis analysis. Namely, our docking model shows that these amino acid residues can form ionic interactions with nifedipine (Fig. 6). Therefore, Phe173^2.52^ and Trp247^6.48^ may be required for the interaction between nifedipine and the A_2B_ receptor. The present docking study also suggested the pharmacophore model. Figure 1 shows the pharmacophore analysis of the calcium antagonists based on the present assay results. The NO group should be essential in the A_2B_ binding and bind the NH of His280^7.43^. The ester parts indicated by the blue circles are common structures binding the OH group of Tyr10^1.35^and the variation of the side chains suggests that these parts should locate toward the outside of the A_2B_ receptor. The –OCH_3_ groups are common structures among these compounds, and are located towards the inside of the receptor, with no additional space. The pharmacophore analysis of the assay results and the docking results are consistent with each other.

Finally, we sought to confirm the *in vitro* results by examining the effect of nifedipine on luminal adenosine-induced intestinal fluid secretion in mice. Specifically, we investigated the effect of adenosine receptor agonists on fluid secretion in the colon. Only BAY60-6583, an A_2B_ receptor agonist, significantly increased intestinal fluid content (Fig. 5b). This finding was confirmed using A_2B_ receptor-deficient mice. These results agreed with those for cAMP accumulation in T84 cells. To our knowledge, this is the first evidence that the colonic A_2B_ receptor is predominantly involved in water secretion into the lumen. We confirmed that adenosine-induced increases in fluid contents were blocked by the selective A_2B_ antagonist PSB-1115 as well as nifedipine, suggesting that the latter’s inhibitory action relied on blocking the A_2B_ receptor. However, A_2B_ receptor in the colon is also expressed in various cell types, such as enteric neurons, vascular cells, and mast cells^18^, which can indirectly contribute to water secretion *in vivo*. Thus, we need to examine this possibility using epithelial cell-specific A_2B_ receptor-deficient mice in our next study. The dose required to inhibit fluid secretion by adenosine was higher for nifedipine than PSB-1115. This is supported by the fact that the affinity of nifedipine for the receptor was lower than that of PSB-1115. However, nifedipine possesses antagonistic calcium activity at a nanomolar level^22^, which may also contribute to the suppression of fluid secretion, a possibility we could not exclude.

Nifedipine is commonly used in medication. In general, anti-diarrhoea drugs can cause constipation in humans. Nifedipine treatment also seems to be associated with constipation^23^. According to adverse events from prescribing information, constipation in nifedipine users has been reported approximately 1% of patients (versus 0% in placebo). Previous reports have showed that nifedipine slows small intestinal transit or suppresses colonic motility in humans^24,25^. Although these results suggest the potential of nifedipine as an anti-diarrhoea agent, its A_2B_ receptor antagonistic activity for intestinal epithelial cells as well as its calcium inhibitory activity in intestinal smooth muscle cells can contribute to the effects.

Our study has also some limitations. First, we did not assess the binding affinity of other adenosine receptor subtypes. We have recently conducted preliminary experiments to determine whether nifedipine binds to A_1_ and A_2A_ receptors. We have found a weak affinity of nifedipine for both receptors (the *K*_i_ values of A_1_ and A_2A_ receptors were 4.3 µM and 4.1 µM, respectively). Thus, we could not fully exclude the involvement of other receptor subtypes in the present study. Although our pharmacological studies using selective agonists indicate the specific involvement of the A_2B_ receptor, experiments using genetically modified cells (e.g., A_2B_ receptor-knockout T84 cells) or mice (e.g., epithelial cell-specific A_2B_ receptor-deficient mice) could be beneficial in future studies. Second, future studies should investigate normal intestinal cell lines, as their receptor expression profiles and responsiveness to adenosine may be different from those of cancer cells. In addition, other types of fluid secretion assays such as the Ussing chamber assay and 2D or 3D secretion assays are necessary to validate our idea. Third, in the present study we did not determine the influence of nifedipine or PSB-1115 on luminal fluid contents in the absence of adenosine treatment. Because A_2B_ receptor can be mainly activated by extracellular adenosine in pathophysiological conditions, we focused on these conditions in this study. However, it is important to investigate whether nifedipine or PSB-1115 affect fluid secretory activity in normal conditions or modulate absorption processes in luminal fluid dynamics, as fluid absorption from epithelial cells occurs before secretion. Here, we observed colonic fluid contents 2 h after adenosine injection. Thus, we could not exclude the possibility that adenosine A_2B_ receptor suppresses absorption.

In summary, the identification of nifedipine, an already approved drug, as an inhibitor of the A_2B_ receptor could facilitate its application in clinical trials as a therapeutic candidate for diarrhoea-related diseases. Moreover, modelling of its interaction with the receptor’s active site could drive the design of specific modifications that ameliorate its function.

## Methods

### Chemicals

Nifedipine, nisoldipine, nitrendipine, nilvadipine, and isradipine were obtained from Tokyo Chemical Industry (Tokyo, Japan) and Wako Pure Chemical Industries (Osaka, Japan). PSB-1115 was purchased from Santa Cruz Biotechnology. Amlodipine besylate (amlodipine), adenosine deaminase (ADA), *N*6-cyclopentyladenosine (CPA), forskolin, and adenosine were purchased from Sigma (St. Louis, MO). CGS21680, IB-MECA, and EHNA hydrochloride (EHNA) were obtained from Abcam (Cambridge, UK). BAY60-6583 was purchased from Tocris Bioscience (Bristol, UK). The compound library consisting of 1,241 existing medicines was a kind gift from LTT Bio-Pharma Co., Ltd (Tokyo, Japan).

### Animals

Male C57/BL6N mice (9–12 weeks old, 22–28 g) and male adenosine A_2B_ receptor (A_2B_AR)-deficient mice were purchased from Charles River Laboratories Japan and Deltagen Inc^26^, respectively. Animals were housed in controlled temperature (22–24 °C) and light (12-h light/12-h dark cycle) conditions for more than 4 days before experiments. The heterozygous mice were backcrossed to C57BL/6 N strain mice for four generations to generate congenic C57BL/6 N strain *ADORA2B* gene mutant mice. Crossbreeding of C57BL/6 N strain *ADORA2B* mutant heterozygous mice generated the same background strain as wild-type (WT) or *ADORA2B* gene knockout homozygous mice. We have confirmed that both WT and C57BL/6 N mice showed similar colonic fluid secretion in normal conditions. The phenotypic properties of the genetically modified mice have also been characterized in previous studies^27–29^. All animal studies were performed in accordance with the ARRIVE^30,31^ and National Institutes of Health guidelines, and were approved by the Animal Care Committees of St. Marianna University (approval numbers: TG180529-5 and 1804015).

### Competitive receptor binding assay

The ligand binding assay was performed using 8-cyclopentyl-1,3-dipropylxanthine,[dipropyl-2,3-^3^H(N)] ([^3^H]-DPCPX). Membrane fractions (20 µg) prepared from human embryonic kidney (HEK) 293 cells expressing human adenosine A_2B_ receptor (Perkin-Elmer) were incubated with 30.5 nM [^3^H]-DPCPX, test drugs, and assay buffer (50 mM HEPES pH 7.4, 5 mM MgCl_2_, 1 mM EDTA) in a 96-well microplate at room temperature for 1 h. A range of concentrations (3 nM to 100 µM) for each compound was tested in triplicate to generate competition curves. The samples were passed through a GF/C filter (Perkin-Elmer) pre-soaked for 1 h with 0.5% BSA and washed four times with wash buffer (5 mM HEPES pH 7.4, 5 mM MgCl_2_, 1 mM EDTA, 0.25% BSA). Filters were then dried for 30 min before attachment to a MeltiLex A (Perkin-Elmer). The radioactivity remaining on the filter was monitored with a MicroBeta Trilux microplate scintillation counter (Perkin-Elmer). Inhibitory binding constant (*K*_i_) values were determined from IC_50_ and K_D_ values according to the Cheng & Prusoff equation^32^. *K*_i_ and IC_50_ values were calculated by non-linear regression analysis using the equation for a sigmoid concentration-response curve (Graph-PAD Prism). The K_D_ value for [^3^H]-DPCPX binding to A_2B_R was determined by saturation assay (K_D_ = 10.9 nM). Non-specific binding was examined in the presence of PSB-1115 (10 µM). All experiments were performed in triplicate. Data were normalized to the results of each compound at 3 nM. The data represent the average of the values from at least three independent experiments.

Test drugs or control drugs (aminophylline, CGS21680, BAY60-6583, PSB-1115, NECA) (approximately 10 µM) were screened using 96-well microplates, each of which included a non-specific binding control (without [^3^H]-DPCPX), buffer control (without drug), and aminophylline and PSB-1115 as positive controls. Before drug screening, we confirmed that the *K*_i_ values of the positive control drugs were similar to those in previous reports and that a selective A_2A_ agonist, CGS21680, had no affinity for the A_2B_ receptor membrane. Each plate was normalized to the buffer control. From the screening, we selected drugs with higher affinity than aminophylline. Then, these candidates were tested at three different concentrations (0.1–10 µM) in triplicate. Finally, we identified 12 candidates that had equivalent binding affinity to aminophylline.

### Calcium mobilization assay

Calcium mobilization was assayed using the FRIPR^®^ calcium-6 assay kit (Molecular Devices) according to the manufacturer’s instructions. Chinese hamster ovary (CHO) cells stably expressing both human adenosine A_2B_ receptor and Gα15 protein (GeneScript) were seeded in a 96-well microplate (poly-D-lysine-coated black plate) at a density of 3×10^4^ cells/well with 100 µL Ham’s F12 containing heat-inactivated 10% FBS, 1% penicillin/streptomycin (Gibco), 400 µg/mL G418, and 100 µg/mL hygromycin. Cells were incubated for 24 h at 37 °C in 5% CO_2_. After that, 100 μL loading buffer containing 2.5 mM probenecid was added to each well, and incubation proceeded for another 2 h. A 20-μL test drug solution was added to the appropriate wells and the plates were kept at room temperature for 10 min. Cells were stimulated by the addition of 20 µL 1 µM adenosine solution or Hank’s balanced salt solution (HBSS) with an injector. Fluorescence intensity (F) was measured by a TECAN plate reader (excitation 485 nm; emission 525 nm; 80 cycles; single detection). Serial concentrations of test drugs dissolved in DMSO were prepared with HBSS (3 to 100 µM). Calcium mobilization (∆F/F) was calculated as follows: ∆F/F = [(maximum F value after adenosine treatment) – (maximum F value before adenosine treatment)] / (maximum F value before adenosine treatment). As these cells overexpress not only the A_2B_ receptor but also the Gα15 protein, this cell line was suitable for the evaluation of A_2B_ receptor binding activity.

### cAMP accumulation assay

Colonic epithelial T84 cells (CCL-248; ATCC) were plated into a 24-well microplate at a density of 2×10^5^ cells/well with 500 μL DMEM/Ham’s F12 (1:1 mixture) supplemented with 5% FBS and 1% penicillin/streptomycin. Cells were incubated for approximately 7 days at 37 °C in 5% CO_2_ until confluent. During preculture, the medium was changed twice a week. Confluent cells were washed with 300 μL HBSS containing 1 unit/mL adenosine deaminase (ADA) before further incubation for 2 h with HBSS without ADA. After that, cells were preincubated with 100 μL 50 µM rolipram for 15 min, and then with 100 μL adenosine receptor agonist and calcium blockers for another 15 min. During adenosine stimulation, ADA inhibitor EHNA (50 µM) was added with rolipram to avoid adenosine degradation. T84 cells were washed with HBSS and lysed with 250 μL 0.1 N HCl at room temperature for 20 min. Intracellular cAMP levels were determined by an enzymatic cAMP assay kit from Cayman Chemical Company (Ann Arbor, MI) according to the instruction manual.

### Assessment of colonic fluid secretion and absorption

To determine the intestinal luminal fluid volume, a mouse intestinal loop model was used as described previously with some modifications^2^. Mice fasted for 18 h were anesthetized with 2% isoflurane. The intestinal tract was exposed using a 1-cm middle incision. The colon was ligated with silk approximately 1 cm distal to the caecal–colon junction. Then 100 μL drug solution or vehicle (saline containing 2.5% DMSO) was injected from the ligated point into the colonic lumen. Immediately, one loop of approximately 2 cm was prepared by ligating the colon around 3 cm distal to the caecal–colon junction. The tract was returned to the peritoneal cavity, and the abdominal incision was closed with sutures. Mice were allowed to recover from the anaesthesia. After 2 h, the colonic loop was removed, and its length and weight (including intraluminal contents) were measured. Then, the loop was opened and wiped to remove residual contents. The volume of residual intraluminal fluid was calculated as follows: Residual fluid volume (μL/cm) = [(isolated loop weight with intraluminal contents) – (isolated loop weight without intraluminal contents)] / loop length. Finally, data were normalized to 100% vehicle. Control mice were treated with saline containing 2.5% DMSO (vehicle) alone. For assessment of the inhibitory activity of nifedipine or PSB-1115 on adenosine-produced colonic water secretion, a 1 mM adenosine solution was used.

### Homology modelling

As no experimental structures of A_2B_ were available at the time, homology-based models were built for A_2B_. These were based on a crystal structure of the closely related A_2A_, which has 55% sequence identity to A_2B_ in the transmembrane region. The crystal structure of A_2A_ (PDB entry 5N2R^33^); complexed with an antagonist (PSB36) served as a structural template. The sequence of human A_2B_ (UniProt entry P29275) was aligned to that of the A_2A_ crystal structure using MATRAS^34^ Subsequently, the alignment in the second extracellular loop (ECL2) was manually edited (Supplementary Fig. 1). The C-terminus was excluded from the model because the coordinates were missing in the template. The cytochrome *b*_562_RIL fused between TM5 and TM6 was replaced with the corresponding wild-type amino acid sequence (QLRTELMD) of A_2B_. According to a mutagenesis study of the four cysteine residues in the ECL2, the C171^45.50^S mutant lost its capacity for high-affinity binding to the A_2B_ antagonist, whereas cysteine-to-serine mutants in the other three positions behaved like the wild-type receptor^35^. Therefore, we considered only the C78^3.25^–C171^45.50^ disulphide bond, which is highly conserved in class A G protein-coupled receptors. The antagonist PSB36 was incorporated and placed in the same position as in the A_2A_ crystal structure. All modelling procedures were performed using Modeller v9.13^36^.

### Docking simulation

The crystal structures of nifedipine found in the Crystallography Open Database^37^ were computationally docked into the A_2B_ model using the sievgene/myPresto docking engine^38^ implemented in MolDesk ver. 1.1^39^. The partial charge was derived by quantum chemical calculations using Gaussian03^40^ at the HF/6-31 G* level followed by RESP fitting^41^. The AMBER99 force field parameters^42^ were applied to A_2B_. All acidic residues (Asp and Glu) and all basic residues (Lys and Arg) were set to be negatively and positively charged, respectively. The His280^7.43^ residue was assumed to be positively charged whereas all other His residues were set to be neutrally charged and protonated at the δ-nitrogen atom. The binding pocket of A_2B_ was specified as a collection of probe points, which were located as the heavy atoms of PSB36 modelled with A_2B_. Flexible docking was performed by twisting the rotatable bonds of nifedipine, while the coordinates of the A_2B_ model remained fixed. The plausibility of docking was evaluated by the scores of the docking program. The best pose giving the highest score is shown in Fig. 6.

### Statistical analysis

Data and statistical analysis were compiled using recommendations on experimental design and analysis in pharmacology^43^. Values obtained from animal experiments were derived from at least two independent experiments. All data are expressed as mean ± standard error of the mean (SEM). For the assessment of colonic fluid secretion, normalization was performed after collection of all data. Statistical analyses were performed using SPSS 24.0 software (IBM). One- or two-way ANOVA followed by Dunnett’s test or Tukey’s test were used to evaluate differences between more than two groups. Differences were considered significant at *P* < 0.05.

## Supplementary information

supplemental file.
