# Supplementary material for: A2B adenosine receptor inhibition by the dihydropyridine calcium channel blocker nifedipine involves colonic fluid secretion
Source: Sci Rep. 2020 Feb 26;10:3555. doi: 10.1038/s41598-020-60147-7 (PMC7044278; doi:10.1038/s41598-020-60147-7)
Supplement: Supplementary file 1 — supplemental file. [file 41598_2020_60147_MOESM1_ESM.docx]

**Supplementary information**

A_2B_ adenosine receptor inhibition by the dihydropyridine calcium channel blocker nifedipine involves colonic fluid secretion

Teita Asano^a*^, Yuto Noda^b^, Ken-Ichiro Tanaka^c^, Naoki Yamakawa^d^, Mitsuhito Wada^e^, Tadaaki Mashimo^f,g^, Yoshifumi Fukunishi^f^, Tohru Mizushima^b*^ and Mitsuko Takenaga^a^

**Supplementary Table 1. Screening results from competition assay**

**
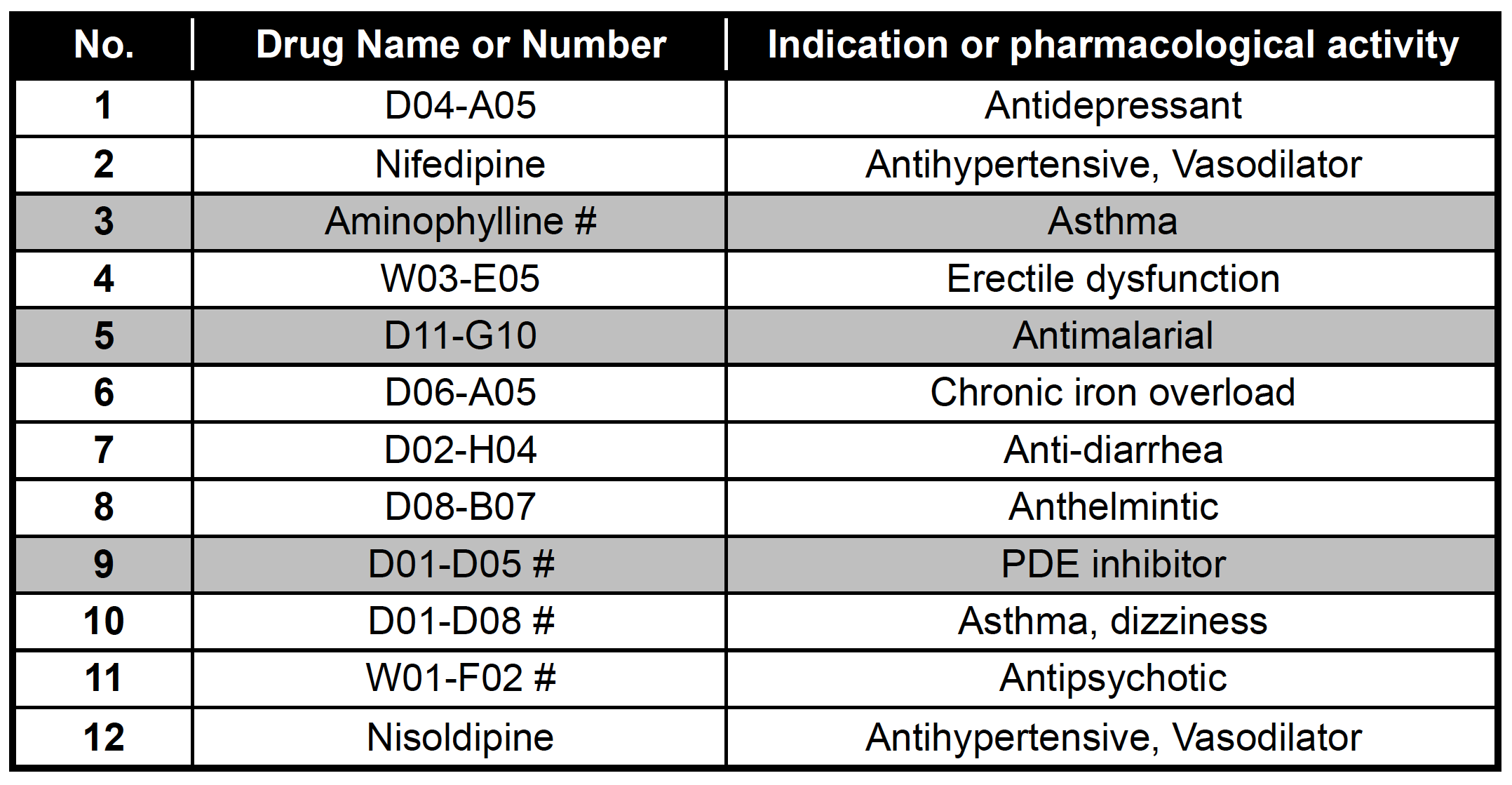
**

Grey layer shows drugs that are already known to have A_2B_ receptor binding affinity.

#, drug with xanthine-like structure.

**Supplementary Fig. 1. Pairwise sequence alignment of the A_2A_ template and A_2B_ receptors.**
